# The influence of neighbourhood formality status and socio-economic position on self-rated health among adult men and women: a multilevel, cross sectional, population study from Aleppo, Syria

**DOI:** 10.1186/1471-2458-13-233

**Published:** 2013-03-16

**Authors:** Balsam Ahmad, Vicky Ryan, Wasim Maziak, Tanja Pless-Mulloli, Martin White

**Affiliations:** 1 Institute of Health & Society, Newcastle University, Richardson Road, Newcastle upon Tyne NE2 4AX, UK; 2 Newcastle Institute for Research on Sustainability, Newcastle University, Newcastle upon Tyne, UK; 3 Robert Stempel College of Public Health and Social Work, Florida International University, Miami, FL, USA; 4 Syrian Centre for Tobacco Studies, Aleppo, Syria; 5 Fuse, UKCRC Centre for Translational Research in Public Health, Newcastle upon Tyne, UK

**Keywords:** Multilevel modelling, Self-rated health, Syria, Formal and informal areas, Neighbourhood, Socioeconomic status, Gender

## Abstract

**Background:**

There is substantial evidence from high income countries that neighbourhoods have an influence on health independent of individual characteristics. However, neighbourhood characteristics are rarely taken into account in the analysis of urban health studies from developing countries. Informal urban neighbourhoods are home to about half of the population in Aleppo, the second largest city in Syria (population>2.5 million). This study aimed to examine the influence of neighbourhood socioeconomic status (SES) and formality status on self-rated health (SRH) of adult men and women residing in formal and informal urban neighbourhoods in Aleppo.

**Methods:**

The study used data from 2038 survey respondents to the Aleppo Household Survey, 2004 (age 18–65 years, 54.8% women, response rate 86%). Respondents were nested in 45 neighbourhoods. Five individual-level SES measures, namely education, employment, car ownership, item ownership and household density, were aggregated to the level of neighbourhood. Multilevel regression models were used to investigate associations.

**Results:**

We did not find evidence of important SRH variation between neighbourhoods. Neighbourhood average of household item ownership was associated with a greater likelihood of reporting excellent SRH in women; odds ratio (OR) for an increase of one item on average was 2.3 (95% CI 1.3-4.4 (versus poor SRH)) and 1.7 (95% CI 1.1-2.5 (versus normal SRH)), adjusted for individual characteristics and neighbourhood formality. After controlling for individual and neighbourhood SES measures, women living in informal neighbourhoods were less likely to report poor SRH than women living in formal neighbourhoods (OR= 0.4; 95% CI (0.2- 0.8) (versus poor SRH) and OR=0.5; 95%; CI (0.3-0.9) (versus normal SRH).

**Conclusions:**

Findings support evidence from high income countries that certain characteristic of neighbourhoods affect men and women in different ways. Further research from similar urban settings in developing countries is needed to understand the mechanisms by which informal neighbourhoods influence women’s health.

## Background

Neighbourhoods matter for the health of their residents and a significant part of the variation in health between neighbourhoods is associated with the neighbourhood context independent of individual characteristics [1,2]. Evidence from high income countries suggests that the type and strength of the relationship between neighbourhood characteristics and health vary by gender.

Reports from Syria and other Arab countries show that Arab women, especially those in low income urban areas bear a disproportionate burden of poor health and disability compared to men [3-8]. These studies have focused on individual determinants of health, and none has considered the relative importance of neighbourhood or other upstream social determinates in explaining health inequalities between men and women. The Arab uprisings have highlighted the potential role of upstream social determinants of health including socioeconomic and political exclusion in fostering discontent, especially amongst the poor [9].

This study fills a gap in the literature on neighbourhoods and health by using multilevel modelling to study the effects of neighbourhood informality and neighbourhood SES on individual health in an urban environment of a developing country. In this paper we use the term ‘informal neighbourhoods’ to denote residential areas built haphazardly with no adherence to urban planning or building regulations. These are neighbourhoods characterised by insecure tenure; illegal or substandard housing structures; poor quality houses and lack of basic services and infrastructure such as potable water or proper sewers. On the other hand, formal neighbourhoods are planned residential areas. The term ‘informal neighbourhoods’ has been used interchangeably in the literature with several other terms such as urban slums, shanty towns, unauthorised settlements or squatter settlements [10]. The UN-HABITAT noted the many variations in the nature of informal settlements between and within countries and sometimes within the same city, and hence acknowledged the difficulty in having a universal definition of these settlements [11]. In Aleppo, Syria more than 40% of the population live in informal neighbourhoods [12]. Differences between these neighbourhoods were noted in the type of industrial workshops, ethnic composition and availability of infrastructure and services [13]. Overall, communities in these settlements, especially women, suffer from a disproportionate burden of ill health and disability [8,13,14].

Evidence of any association between health, neighbourhood informality and neighbourhood socioeconomic status (SES) is important for understanding geographical health inequalities and hence has implications for health and social policies targeting neighbourhoods. A considerable proportion of the urban poor live in informal settlements in developing countries and hence it is important to consider the role of neighbourhood informality status on health.

Using data from a large household survey in Aleppo, Syria, we compare neighbourhood variation in SRH among adult men and women (age 18–65 years) residing in formal and informal neighbourhoods in Aleppo, and investigate whether neighbourhood SES and formality status are associated with SRH after adjusting for individual characteristics.

## Methods

### Setting

Residential neighbourhoods in Aleppo were defined as either ‘formal’ or ‘informal’. There were 87 neighbourhoods in the formal zone and 29 neighbourhoods in the informal zone, as described in the municipal registry. The population of Aleppo was split between the formal and informal zones in a ratio of approximately 2:1. The number of households within a neighbourhood varied from 97 to 18,100.

### Design of the study

Data were from the Aleppo Household Survey (AHS), which was conducted by the Syrian Centre for Tobacco Studies (SCTS) in 2004. The survey combined a health interview with a health examination, providing a ‘baseline map’ of the main health issues and exposures affecting communities in Aleppo. Details of the survey, population and sampling have been described elsewhere [8,15] but relevant details on the structure of the dataset are given here. Ethical approval for the survey study was obtained from Institutional Review Boards of the university of Memphis in the USA and the SCTS [15].

The survey design employed multistage random sampling. The sampling unit was the household with one person aged 18–65 randomly selected from each household to participate. Households were clustered in neighbourhoods within two zones: formal and informal. Sampling was within zone, with a target of 1000 households from each. Firstly, within each zone, neighbourhoods were randomly selected with probability proportional to the population size of the neighbourhood. A fixed proportion of households from each neighbourhood was then selected by first choosing a random starting point followed by the systematic sampling of every fifth household until the neighbourhood target sample size was achieved (0.6% of households per neighbourhood in the formal zone and 0.9% in the Informal zone). The resultant sample of households would be spatially closer together than one would expect by chance if simple random sampling within administrative neighbourhood boundary had been employed.

### Variables used in the analysis

Table 1 includes a description of the outcome variable and compositional (individual-level) and contextual (neighbourhood-level) variables included in the analysis. In the absence of neighbourhood data from the Census in Aleppo, we aggregated the individual level socioeconomic indicators from the survey data to the neighbourhood level [16].

**Table 1:** Variables used in the analyses

**Type of variable**	**Original measurement**	**Used in the analysis**	**Other information**
**Outcome variable**			
Self-rated health (SRH)	Question “in general, how do you describe your overall health?” were on a 5-point scale: excellent/good/normal/bad/very bad	Grouped into three categories: excellent (excellent and good), normal, and poor (bad and very bad)	
**Individual-level variables**			
***Socio-demographic***			
**Age**	Years		
**Marital Status**	Four levels: Single, Married, divorced, widowed	Binary**:** never married (single) and ever married (including married, widowed and divorced)	
***Individual level socio-economic status***			
Education level	Illiterate	Categorised as: illiterate, <=9 years of education and >9 years of education	Nine years signifies compulsory basic education in Syria
	Total school years finished		
Employment	Student or full-time house wife (economically inactive), employee (government or other) and employer (private business or self-employed professional)		
Car ownership	Private ownership of a car was included as a binary variable, either yes or no		
Item ownership	Binary (yes=1, no=0) for 6 items: a telephone, a mobile phone, a personal computer, an air conditioner, a television and a satellite dish	Numerical variable: summed responses to questions on the ownership of six items (yes=1, no=0) to create a numerical variable ranging from zero (no items) to six	
Household crowding	Number of people living in the house	Divided the number of people living in the house by the number of rooms	
	Number of rooms in the house		
***Health and Health behaviours***			
Body Mass Index (BMI)	Weight in kilograms divided by the square of the height in meters (kg/m^2^).		
Level of physical activity	“Do you practice sport regularly?”		
Smoking status	**“**Do you smoke cigarettes daily, occasionally or not at all”	Binary: currently smoking (daily or occasionally) or not currently smoking at all	
Social support	“Do you have someone who supports you when needed?“ (yes, no)		
	“Do you have someone to share happiness and sorrow with? (yes, no)		
***Perception of the neighbourhood***			
Annoyance of outdoor air pollution	“How much are you annoyed by outdoor air pollution if you keep the windows open?” (Not at all, somehow, much, very)		
Annoyance of outdoor air pollution and noise	“How much are you annoyed by outdoor noise if you keep the windows open?” (Not at all, somehow, much, very)		
**Neighbourhood-level variables**			
***Neighbourhood level SES***			
Percent illiterate			Individual level variables SES from the survey data were aggregated to the neighbourhood level
Percent unemployed			
Average number of items owned			
Percent with high density housing			
Percent with no car			
***Formality Formal/Informal***			These zones were non-overlapping with the formal zone occupying the west of Aleppo and the informal zone spreading out along the northern, eastern and southern borders of the formal zone. Formality was included as a binary fixed effect variable in the analysis with “formal” being the reference category

### Statistical analysis

Multilevel models were fitted in MLwiN software (version 2.02) [17] to account for the hierarchical structure of the data. Models were fitted with and without design based weights. Design based weights were calculated to adjust for the unequal probability of selection of individuals in the sample. Neighbourhood and individual level weights were calculated separately and incorporated into the multilevel analysis at the appropriate level [18]. Design weights were calculated for the whole sample of 2038 individuals, based on Census data for the combined male and female population of Aleppo as no information was available by gender. For most variables, inferential conclusions were very similar for the weighted and un-weighted analyses. However, there were some discrepancies for categorical variables when cell frequencies were small; in such cases un-weighted findings are reported [19]. We report un-weighted multilevel binary logistic regression models with excellent SRH as the reference category. Multilevel models were fitted using Markov Chain Monte Carlo (MCMC) estimation [20] with orthogonal parameterisation and parameter expansion [21]. We compared successive models using the deviance information criterion [22]. Inferences were drawn from chains of length 50,000 after a burn-in of 2,000 with the means and 95% CIs from the posterior distribution given in the results.

The variance between neighbourhoods was expressed as both an intra-class correlation coefficient (ICC) and a median odds ratio (MOR). The ICC was calculated assuming a linear threshold model [23] and can be interpreted as the percentage of the unexplained variation in the probability of poor/normal SRH, which can be attributed to differences between neighbourhoods. The MOR [24] is an alternative way of expressing neighbourhood level variation that translates the variance to the odds ratio (OR) scale, making the MOR comparable with the ORs of individual or neighbourhood level variables. An MOR of one indicates no residual variation between neighbourhoods in the probability of poor/normal SRH.

The analysis proceeded in four stages, separately for males and females. Firstly, we fitted an empty variance components model examining the amount of variation in the probability of poor/normal SRH that could be attributed to the individual and neighbourhood levels of analysis.

The second stage expanded the empty MLM by including age, BMI, smoking status, level of physical activity, marital status, social support variables and perception of neighbourhood variables as fixed effects. Formality was also added at this stage as a fixed effect. In addition to main effects, all plausible interactions between pairs of individual level variables and between individual level variables and formality were explored. The significance of fixed effects terms in the model were tested by considering whether the odds ratio differed significantly from one. To assess whether there was an association between individual level SES and SRH after adjusting for other variables, each individual level SES variable was then added in turn and simultaneously. In the third stage, we allowed any statistically significant fixed effect for individual level SES to vary randomly across neighbourhoods, enabling us to determine whether individual SES variables predicted SRH in a manner that differed across neighbourhoods. Finally, we entered the aggregated neighbourhood level SES variables one at a time and assessed their inclusion on the regression coefficient for individual level SES and on the between neighbourhood variation.

Analyses were also performed with and without very small and very large neighbourhoods to assess the influence of neighbourhood size on the fixed effect estimates. Neighbourhood size was also included as an explanatory variable in an attempt to determine if there was anything ‘different’ about small neighbourhoods. MCMC diagnostics were used to assess parameter estimation and multilevel residuals were examined to assess model fit.

## Results

### Sample characteristics

Of 2370 people invited, a total of 2,038 (86%) people took part in the survey. Survey respondents were clustered in 45 neighbourhoods; 1017 respondents from 27 formal neighbourhoods and 1021 respondents from 18 informal neighbourhoods. The sample included 921 (45.2%) men and 1117 (54.8%) women. For females, the median achieved number of respondents per neighbourhood was 22, range 2 to 95 (inter-quartile range 9 to 35); for males, the median was 17, range 1 to 72 (inter-quartile range 10 to 27).

Table 2 shows a simple summary of the sample characteristics by formality and gender.

**Table 2:** Self-reported sample characteristics by formality and gender

		**Formal**		**Informal**		**Both**	
**Variables**	**Level**	**Men (n=451)**	**Women (n=566)**	**Men (n=470)**	**Women (n=551)**	**Men (N=921)**	**Women (N=1117)**
**Categorical variables**		**n (%)**	**n (%)**	**n (%)**	**n (%)**	**N (%)**	**N (%)**
Self-rated health	Bad/v.bad	21 (4.7)	57 (10.1)	36 (7.7)	72 (13.1)	57 (6.2)	129 (11.5)
	Normal	151 (33.5)	239 (42.2)	123 (26.2)	212 (38.5)	274 (29.8)	451 (40.4)
	Excellent/good	279 (61.9)	270 (47.7)	311 (66.2)	267 (48.5)	590 (64.1)	537 (48.1)
Marital status	Never married	133 (29.5)	122 (21.6)	72 (15.3)	104 (18.9)	205 (22.3)	226 (20.2)
	Ever Married	318 (70.5)	444 (78.4)	398 (84.7)	447 (81.1)	716 (77.7)	891 (79.8)
Education	Illiterate	26 (5.8)	90 (15.9)	102 (21.7)	207 (37.6)	128 (13.9)	297 (26.6)
	<9 years	232 (51.4)	286 (50.5)	314 (66.8)	299 (54.3)	546 (59.3)	585 (52.4)
	>9 years	193 (42.8)	190 (33.6)	54 (11.5)	45 (8.2)	247 (26.8)	235 (21.0)
Employment	Unemployed	62 (13.7)	465 (82.2)	33 (7)	499 (90.6)	95 (10.3)	964 (86.3)
	Employee	190 (42.1)	81 (14.3)	239 (50.9)	32 (5.8)	429 (46.6)	113 (10.1)
	Employer	199 (44.1)	20 (3.5)	198 (42.1)	20 (3.6)	397 (43.1)	40 (3.6)
Crowding	High	27 (6)	48 (8.5)	182 (38.7)	208 (37.7)	209 (22.7)	256 (22.9)
	Medium	110 (24.4)	130 (23.0	167 (35.5)	193 (35.0)	277 (30.1)	323 (28.9)
	Low	314 (69.6)	388 (68.6)	121 (25.7)	150 (27.2)	435 (47.2)	538 (48.2)
Smoking	Not smoking	187 (41.5)	418 (73.9)	179 (38.1)	434 (78.8)	366 (39.7)	261 (23.4)
	Currently smoking	264 (58.5	148 (26.1)	290 (61.7)	113 (20.5)	554 (60.2)	852 (76.3)
Practice sport regularly	No	348 (77.2)	477 (84.3)	397 (84.5)	495 (89.8)	745 (80.9)	972 (87.0)
	Yes	103 (22.8)	89 (15.7)	73 (15.5)	56 (10.2)	176 (19.1)	145 (13.0)
*Social support*							
Has someone to support &help	No	94 (20.8)	78 (13.8)	115 (24.5)	99 (18.0)	209 (22.7)	177 (15.8)
	Yes	357 (79.8)	488 (86.2)	355 (75.5)	452 (82.0)	712 (77.3)	940 (84.2)
Has someone to share happiness & sorrow	No	41 (9.1)	51 (9.0)	54 (11.5)	78 (14.2)	95 (10.3)	
	Yes	410 (90.9)	515 (91.0)	416 (88.5)	473 (85.8)	826 (89.7)	988 (88.5)
Outdoor air pollution	Not at all	186 (41.2)	185 (32.7)	192 (40.9)	155 (28.1)	378 (41)	340 (30.4)
	Somewhat	160 (35.5)	209 (36.9)	180 (38.3)	184 (33.4)	340 (36.9)	393 (35.2)
	A lot	105 (23.3)	172 (30.4)	98 (20.9)	212 (38.5)	203 (22)	384 (34.4)
Outdoor noise	Not at all	98 (21.7)	118 (20.8)	152 (32.3)	118 (21.4)	250 (27.1)	236 (21.1)
	Somewhat	150 (33.3)	177 (31.3)	167 (35.5)	189 (34.3)	317 (34.4)	366 (32.8)
	A lot	203 (45.0)	271 (47.9)	151 (32.1)	244 (44.3)	354 (38.4)	515 (46.1)
**Continuous variables**		**Mean ± SD**	**Mean ± SD**	**Mean ± SD**	**Mean ± SD**	**Mean ± SD**	**Mean ± SD**
Age (years)		37.1 ± 12.4	36.1 ± 12.2	35.8 ± 12	32.5 ± 11.3	36.4 ± 12.2	34.3 ± 11.9
Item ownership (0–6)		3.5 ± 1.4	3.3 ± 1.4	3.5 ± 1.4	2.3 ± 1.1	2.82 ± 1.42	2.8 ± 1.3
Body mass index (BMI)		27.7 ± 5	36.4 ± 7.2	27.1 ± 5.1	29.7 ± 6.8	27.4 ± 5.1	30 ± 7

Overall, women were more likely to describe their health as poor (11.5% of those reporting poor SRH were women compared to 6.2% of men). They were also more likely to report that they were illiterate (26.6% of women compared with 13.9% of men) and more likely to be unemployed (86.3% of women reported they were unemployed compared with 10.3% of men).

Tables 3 and 4 present the results of fitting the empty and final variance component models for females and males separately. The final model results are presented as odds ratios together with 95% CIs (also adjusted for age, BMI, smoking status and level of physical exercise). For the empty models the ICCs suggest there is some evidence of variation in the probability of poor/normal SRH between neighbourhoods. For females 6% of the variation in the probability of both poor and normal SRH (versus excellent) is attributable to neighbourhoods and for males 7% of the poor SRH and 4% of the normal.

**Table 3:** **Estimates of the between neighbourhood variance in SRH**^**1**^

	**Males**		**Females**	
	**Poor Versus excellent**	**Normal versus excellent**	**Poor versus excellent**	**Normal versus excellent**
	**(n=647)**	**(n=864)**	**(n=666)**	**(n=988)**
**Random effects**				
Variance between neighbourhoods (95% CI)	0.25 (0.002 – 0.96)	0.15 (0.01 – 0.39)	0.20 (0.001 – 0.68)	0.21 (0.05 – 0.47)
ICC	0.07	0.04	0.06	0.06
MOR (95% CI)	1.60 (1.04 –2.54)	1.44 (1.08 – 1.80)	1.53 (1.03 –2.18)	1.54 (1.22 – 1.92)

^1^ Based on the empty models for males and females.

**Table 4:** Odd ratios (95% CIs) and measures of residual neighbourhood variance for the final multilevel binary logistic regression models (adjusted for age, BMI, smoking status, and level of physical exercise)

	**Males**		**Females**	
	**Poor Versus excellent**	**Normal versus excellent**	**Poor versus excellent**	**Normal versus excellent**
	**(n=647)**	**(n=864)**	**(n=666)**	**(n=988)**
	**OR (95% CI)**	**OR (95% CI)**	**OR (95% CI)**	**OR (95% CI)**
**Fixed effects**				
***Individual level***				
Marital status				
Ever married	1.0	1.0	1.0	1.0
Never married	3.2 (1.2, 8.9)	1.2 (0.7, 1.9)	0.2 (0.1, 0.5)	0.6 (0.4, 0.9)
**Social support**				
Has someone to share happiness and sorrow	1.0	1.0	1.0	1.0
No	1.9 (0.7, 6.0)	0.9 (0.5, 1.6)	0.3 (0.2, 0.7)	0.8 (0.5, 1.3)
Yes				
Has someone to support and help	1.0	1.0	1.0	1.0
No	0.4 (0.2, 0.9)	1.0 (0.7, 1.5)	0.8 (0.4, 1.5)	1.2 (0.8, 1.9)
Yes				
**Perception of neighbourhood**				
Suffered outdoor noise				
a lot	1.0	1.0	1.0	1.0
Somewhat	0.4 (0.2, 0.9)	0.6 (0.4, 0.9)	0.9 (0.5, 1.5)	0.9 (0.6, 1.2)
Not at all	0.2 (0.1, 0.6)	0.4 (0.3, 0.7)	0.5 (0.3, 1.0)	0.7 (0.4, 1.0)
Suffered outdoor air pollution				
a lot	1.0	1.0	1.0	1.0
Somewhat	0.5 (0.2, 1.1)	0.9 (0.6, 1.3)	0.6 (0.3, 1.0)	0.9 (0.6, 1.2)
Not at all	0.4 (0.2, 1.1)	0.7 (0.4, 1.1)	0.6 (0.3, 1.1)	0.8 (0.5, 1.2)
**Individual SES **^**1**^				
Item ownership (for an	0.9 (0.7, 1.3)	1.0 (0.9, 1.2)	0.7 (0.6, 0.9)	0.9 (0.8, 1.0)
increase of 1 item)				
***Neighbourhood level***				
**Formality**				
Live in the formal zone	1.0	1.0	1.0	1.0
Live in the informal zone	1.4 (0.3, 5.4)	0.6 (0.3, 1.2)	0.4 (0.2, 0.8)	0.5 (0.3, 0.9)
**Neighbourhood SES**^**1**^				
Neighbourhood average item ownership (for an increase of one item on average)	0.8 (0.3, 2.0)	0.8 (0.5,1.3)	0.4 (0.2, 0.8)	0.6 (0.4, 0.9)
**Random effects**				
Variance between neighbourhoods (95% CI)	0.61(0.01, 1.96)	0.17 (0.02, 0.44)	0.08 (0.00, 0.38)	0.20 (0.03, 0.49)
ICC	0.16	0.05	0.02	0.06
MOR (95% CI)	2.10 (1.11 –3.78)	1.48 (1.14 – 1.88)	1.32 (1.00 –1.79)	1.53 (1.18 – 1.95)

^(1)^ All five individual SES variables were considered one at a time and simultaneously both at the individual level and the neighbourhood level. In the final model for females, only item ownership remained statistically significant when more than one SES variable was included. For males, none were statistically significant in the multivariable model, but item ownership has been included for comparison.

For females, of the five separate individual level SES variables, only ‘number of household items owned’ was significantly associated with SRH after adjustment for all other variables in the model. For an additional item in the home, females were less likely to have poor or normal SRH versus excellent. The individual level ORs for item ownership were attenuated only very slightly when neighbourhood level average item ownership was included in the models, thus suggesting a contextual effect of neighbourhood average item ownership independent of individual level ownership. Furthermore, after adjusting for neighbourhood SES a woman residing in the informal zone was less likely to report poor or normal SRH than excellent suggesting a protective effect of informality on health (all else being equal).

For males, no individual level or neighbourhood level SES variables were significantly associated with SRH after having adjusted for all other variables in the models. However, men who reported that they suffered at home from outdoor noise ‘a lot’ compared with ‘somewhat’ or ‘not at all’ were more likely to have poor or normal SRH versus excellent (see Table 4).

## Discussion

To our knowledge, this is the first multilevel modelling study from any developing country to study the effects of neighbourhood formality and neighbourhood SES on self-rated health in trying to understand health inequalities between men and women. We found little evidence of clustering of poor SRH at the neighbourhood level. However, neighbourhood socioeconomic status (as measured by the number of household items owned) and formality were associated with the SRH of women but not men.

We found that as a proportion of the total variation, the variation between neighbourhoods in poor/normal self-rated health for females and males was small and non-significant. This is similar to what has been observed in other multilevel studies from western countries [2].

We found that one measure of neighbourhood socioeconomic status (average number of household items owned) to be associated with SRH, as shown in previous research [1,2]. However, only in females were the relationship between neighbourhood average item ownership and SRH statistically significant. The results from this study support the notion that the place where a person lives in Aleppo affects men and women in different ways similar to high income countries [25-29]. For example, in the UK Stafford et al.(2005) found that a range of neighbourhood characteristics, such as the socio-political environment, amenities, the physical environment and economic characteristics were more consistently associated with women’s self-rated health than of men’s [29]. However, the results here support results from an earlier study from Aleppo on individual determinants of SRH in men and women, which showed that individual socioeconomic status was a more important predictor of SRH in women compared to men [3].

We postulate that the gender differences observed in this study in the health effects of neighbourhood SES can be explained by gender roles. In turn, gender roles in Aleppo are influenced by the culture of the largely traditional Muslim society, where the segregation of men and women is paralleled by a separation of social knowledge and the way they occupy their environment. Women in this setting are more likely to be economically inactive and are more likely to spend time in their home and neighbourhood doing domestic work. This in turn makes them more vulnerable to the effects of their local environment [28,29]. Additionally, our results show that formality was a significant predictor of women’s but not men’s self-rated health. All things being equal, a woman was more likely to have poor SRH if she lived in a formal neighbourhood than in an informal neighbourhood. It is important to understand the mechanisms behind this finding, given the poor environmental quality and limited neighbourhood resources and services in informal areas [8,13]. Is there something else in the characteristics of informal areas that make them protective of women’s self-rated health? The neighbourhoods that make up the informal zones in this study tended to be poorer with a relatively small spread of average household items, median 2.3, range 1.5 to 2.7 (inter-quartile range 2.0 to 2.4); whereas the neighbourhoods in the formal zone tended to be wealthier but with a wider spread of average items, median 3.0 range 2.0 to 5.4 (inter-quartile range 2.8 to 3.6) but with only 2 formal neighbourhoods with <2.5 items on average. Therefore, formality and neighbourhood SES, as measured by average item ownership, are closely related but remain independent measures (i.e. there is a part of formality that is not explained by neighbourhood SES). Could it be that neighbourhood formality is measuring another neighbourhood characteristic that has more influence on women’s health? Or is it that the larger socioeconomic inequality in formal areas compared to the informal exerts an influence on women’s health? These are questions that need to be addressed in further qualitative research from this setting.

### Limitations and strengths

Firstly, this is a secondary analysis of cross-sectional data and hence it was not possible to consider changes over time in the explanatory or outcome variables. It therefore potentially suffers from the problem of self-selection, whereby people are selected into residential areas based on unmeasured individual characteristics that are relevant to health [30,31]. Findings from previous studies in the West show that people’s ratings of health depends on current health status as well as on changes over time in relation to socioeconomic status, chronic disease, functional disability and mental health [32,33]. Although the AHS included information on functional disability and chronic disease, these variables were not included in our analysis since these are measures which are highly correlated with self-rated health. Additionally, cross-sectional surveys do not include longitudinal and historical data on neighbourhoods which interact with, and are heavily influenced by, a multitude of macro-level factors such as social, cultural, economic and policy contexts of states [34,35]. They also change over time as a result of societal processes such as economic cycles or demographic shifts and migration [36]. This is a particular issue for informal areas, which may change on a day to day basis. The study also lacked data on neighbourhood SES which are not available in Syria. In the absence of such information the importance of contextual SES has been assessed by aggregating individual level indicators from the survey data to the neighbourhood level. For some neighbourhoods, aggregate SES measures were therefore based on the responses of only a few participants (as noted in the Results section, the lower quartile for achieved neighbourhood size was 9 for females and 10 for males). Such averaging of data from very few respondents might have introduced measurement error into the estimation of our neighbourhood level estimates. To explore the impact of these ‘small’ neighbourhoods on the estimation of the fixed effects at the neighbourhood level, the analyses were repeated omitting neighbourhoods with <10 respondents. The resultant final model ORs for neighbourhood level average item ownership were the same to one decimal place. Other authors have encountered similar numerical issues [37]. Those authors also observed a stronger relationship between an individual outcome variable and an aggregated contextual level variable than between the outcome variable and the corresponding individual level variable (as we do here for self-reported health and neighbourhood average item ownership among females). We conclude, like them, that this may represent a genuine contextual effect. However, further research via simulation studies, is needed to investigate the utility of aggregated neighbourhood level variables as measures of contextual effects. A further limitation of this study is the low statistical power to estimate between neighbourhood variation, mainly as a result of the small number of individuals available within a high proportion of the neighbourhoods [31,38,39]. This may explain why as a proportion of the total variation, variation between neighbourhoods in poor/normal self-rated health for females and males was estimated from the model to be small and non-significant.

Despite these limitations, this study is unique in that it considers neighbourhood informality status which is a relevant neighbourhood characteristic for the health of urban residents in developing countries. Therefore, by using an integral measure of neighbourhood (defined as a feature of an area only measurable at an ecological level of neighbourhood) this study has avoided relying solely on a derived neighbourhood measure such as SES which is based on the aggregate characteristics of individuals [36].

Additional strengths of this study include the way in which neighbourhoods were operationalised. Whist formal and informal areas correspond to administrative units that are defined by the local government, the way the neighbourhoods were measured corresponds to smaller, more homogenous clusters or units that are more likely to match people’s perceptions of their local neighbourhoods and hence could be more related to the way they rate their health [2,35]. Another strength of this study was the inclusion in the analysis of correlates of SRH, such as social support, physical activity and smoking status, that have been found to vary by gender in Aleppo [3].

## Conclusions

Our study is the first to consider neighbourhood socioeconomic status and informality status in studying the relationship between neighbourhood and health. We did not find evidence of clustering at a neighbourhood level. However, our findings show that living in an informal neighbourhood was associated with better health for women but not men. There is a need for more neighbourhood and health studies from other similar settings to replicate these findings. There is also a need for studies to understand the process by which informality of neighbourhoods influence women’s health. This could be done in multilevel studies by using other neighbourhood characteristics such as measures of social capital and studying their relationship with health outcomes by gender or by using qualitative methods to uncover the mechanisms by which neighbourhood informality influence women’s health. We recommend that gender and neighbourhood informality status should be taken into account when designing interventions to improve health in urban settings in developing countries. Our study adds to the body of evidence on gender, urban and health inequalities in developing countries. Understanding these influences in the Syrian context will be particularly important when the country emerges from the current violent conflict.

## Abbreviations

AHS: Aleppo household survey; BMI: Body mass index; ICC: Intra-class correlation coefficient; MCMC: Markov Chain Monte Carlo; SCTS: Syrian Centre for Tobacco Studies; SES: Socioeconomic status; SRH: Self-rated health.

## Competing interests

The authors declare that they have no competing interests.

## Authors’ contributions

BA designed the study, contributed to statistical analysis, led writing of the manuscript, interpretation of results and manuscript revision. VR undertook the multilevel modelling, drafted the methods and results sections, contributed to the interpretation of the results and commented on successive drafts of the manuscript. WM, TPM and MW contributed to study design and data interpretation, and commented on successive drafts of the manuscript. All authors approved the final version of the manuscript.

## Pre-publication history

The pre-publication history for this paper can be accessed here:

http://www.biomedcentral.com/1471-2458/13/233/prepub
